# Interest in Continued Use After Participation in a Study of Over-the-Counter Progestin-Only Pills in the United States

**DOI:** 10.1089/whr.2022.0056

**Published:** 2022-11-09

**Authors:** Kate Grindlay, Katherine Key, Carmela Zuniga, Alexandra Wollum, Kelly Blanchard, Daniel Grossman

**Affiliations:** ^1^ Ibis Reproductive Health, Cambridge, Massachusetts, USA.; ^2^ Ibis Reproductive Health, Oakland, California, USA.; ^3^ Advancing New Standards in Reproductive Health (ANSIRH), Bixby Center for Global Reproductive Health, Department of Obstetrics, Gynecology, and Reproductive Sciences, University of California, San Francisco, Oakland, California, USA.

**Keywords:** adolescent and pediatric gynecology, contraception, contraceptives, oral, family planning, health policy, nonprescription drugs

## Abstract

**Objective:**

To assess interest in continued use of over-the-counter progestin-only pills among individuals who used them in a trial.

**Methods:**

From January 2020 to September 2021, we conducted a cross-sectional online survey with individuals who completed participation in a trial evaluating over-the-counter use of norgestrel 0.075 mg tablets in the United States. We calculated descriptive statistics, Pearson's chi-square and Fisher's exact tests, and logistic regression models to assess likelihood of future over-the-counter progestin-only pill use, reasons for interest/noninterest, situations for over-the-counter progestin-only pill use, willingness to pay for an over-the-counter progestin-only pill, likelihood of future preventive health screenings, prior difficulties getting prescription contraception, and background characteristics.

**Results:**

Among 550 adult and 115 adolescent participants (75% response rate), 83% reported likelihood of future over-the-counter progestin-only pill use. Hispanic/Latinx and Black participants and adults with public insurance, prior pregnancies, and some college reported higher likelihood of future use compared with their counterparts. Among likely users, 90% were interested in long-term use and 79 % ≥ 25 years of age reported they would get future preventive screenings; participants would pay up to $20/month on average. Primary reasons for interest included convenience (81%), ease of access (80%), and saving time (77%) and money (64%). The primary reason for noninterest was bleeding associated with progestin-only pill use (52%).

**Conclusion:**

There was high interest in continuing to use over-the-counter progestin-only pills among individuals who had used them in a study. These findings highlight the real-world acceptability of taking a progestin-only pill without a prescription, and contribute to evidence supporting over-the-counter access.

## Introduction

Oral contraceptives have been available in the United States only by prescription since their approval in 1960; however, they are available without a prescription in more than 100 countries,^1,2^ including Mexico. Research has shown that women living in the United States on the Mexico border who access oral contraceptives without a prescription in Mexico choose to do so because of cost and not having to go to a doctor and are largely satisfied with their method source.^3^ Studies show that people can self-screen for contraindications to oral contraceptives using a simple checklist,^4,5^ and oral contraceptives are not addictive or toxic if overdosed.^6^ Individuals accessing them over the counter have greater continuation rates compared with those getting them by prescription^7^ and continue to get preventive health screenings.^8^

The work to complete an application to the United States Food and Drug Administration (FDA) to make a progestin-only pill containing 0.075 mg norgestrel available over the counter in the United States is underway.^9,10^ While the pathway to over-the-counter sale may be easier for progestin-only pills than combined oral contraceptives given their fewer and rarer contraindications,^5^ progestin-only pills are not widely used in the United States. Currently, only two formulations of progestin-only pills are marketed in the United States, and one study estimated that only 4% of oral contraceptive users were taking a progestin-only pill.^11^

As part of the application to the FDA to make 0.075 mg norgestrel tablets available over the counter in the United States, the sponsor, HRA Pharma, conducted the Adherence with Continuous Dose Oral Contraceptive: Evaluation of Self-Selection and Use (ACCESS) study, an interventional, phase III, multicenter, open-label self-selection, and actual use trial.^10^ The objective of the ACCESS study was to assess whether consumers 11 years of age and older selected and used norgestrel 0.075 mg tablets in a manner consistent with package directions in an over-the-counter setting for up to 6 months.^10^ The ACCESS study included 683 adult women ages 18 and older and 200 adolescents 11–17 years of age who used the norgestrel pill during the study.

We conducted a follow-up survey with ACCESS study participants after they had completed their trial participation. The objective of our study was to assess interest in continued use of over-the-counter progestin-only pills among individuals who used them in a trial. This study provides the first data on experiences using a progestin-only pill in an over-the-counter environment in the United States and on interest in continued use among this population, and will help inform a successful launch of this product.

## Materials and Methods

From January 2020 to September 2021, we conducted a cross-sectional online survey with individuals who completed ACCESS study participation. Inclusion criteria for our follow-up study included being a participant who had closed out of the ACCESS study, having either completed the full 6-month study participation or who elected to discontinue study participation at an earlier time. We aimed to include all individuals who had completed ACCESS study participation, with a minimum sample size of 164 participants that would enable us to estimate with 80% power and a ± 5% margin of error the proportion of participants who would continue use of an over-the-counter progestin-only pill if available in the future, our primary outcome of interest.

Upon closing out of the ACCESS study, each individual was assigned a unique study ID to participate in our survey. Study IDs were used to help ensure that only individuals who participated in the ACCESS study could take part, and to prevent respondents from taking the survey more than once. No identifying information from the ACCESS study was provided to our study team.

The ACCESS study team gave invitations to participate in our follow-up study through recruitment postcards or verbally during in-person and phone end-of-study ACCESS visits, and with two invitation/reminder emails. Recruitment materials contained the subject's unique follow-up study ID and our survey website link. Participants were told the anticipated time to complete the survey, compensation amount, and that our anonymous survey aimed to learn more about their opinions on the pill they had used during the ACCESS study.

Our study was approved by the Allendale Investigational Review Board. Before being directed to the follow-up survey, all adults completed an informed consent form and minors completed an assent form. Because the research posed minimal risk to minors, we received a waiver of parental permission under 45 CFR 46.408(c).^12^

Data were collected in an online survey that included 42 questions using Qualtrics (Provo, UT). The survey included questions on participants' likelihood of future over-the-counter progestin-only pill use, reasons for interest/noninterest, situations in which they would use an over-the-counter progestin-only pill, the highest price interested participants would pay per month, likelihood of future preventive health screenings, prior difficulties getting prescription birth control, and background characteristics. After completing the survey, participants were compensated with a $25 Amazon or Starbucks gift card of their choosing.

Over-the-counter access was described to participants as follows: “Right now, you need a prescription from a doctor or nurse to get birth control pills. But it could be possible for people to get birth control pills ‘over the counter’ without a prescription. With ‘over-the-counter’ access, birth control pills would be available on the shelf at a pharmacy or grocery store just like cough medicine or some allergy pills. You would not need a prescription from a doctor or nurse. You would not need to talk to anyone about buying birth control pills (not a doctor, pharmacist, or parent) unless you wanted to. If you had a question, you could talk to a pharmacist.”

We assessed participants' likelihood of future over-the-counter progestin-only pill use with the following question: “How likely are you to buy and use [the progestin-only pill you used in the ACCESS study] if it is available over the counter, without a prescription?” We categorized people as “likely” to use a future over-the-counter pill if they reported being “very likely” or “somewhat likely” (vs. “somewhat unlikely,” “very unlikely,” “not sure,” or “prefer not to answer”).

We assessed situations in which likely participants would use an over-the-counter progestin-only pill and their reasons for interest/noninterest (among likely/not likely participants, respectively) with categorical questions that included a set of response options derived from prior research^13,14^ and an “other—specify” option; participants could select more than one response. We also invited participants to tell us more about their reasons for interest/noninterest in optional open-response text.

We assessed the highest price interested participants would pay per month for an over-the-counter progestin-only pill with the following open-response question: “What is the highest price (in dollars) that you would pay for each month's supply of [the progestin-only pill you used in the ACCESS study] if it were available over the counter in a pharmacy or store without a prescription?” We coded the highest price interested participants would pay as a categorical variable ($0, $1–10, $11–20, $21–30, >$30, missing), and calculated the mean and median highest price among participants who were willing to pay >$0.

To assess screening history, we asked participants ≥25 years of age whether they had had a cervical cancer screening in the prior 3 years. We assessed likelihood of getting future preventive health screenings through the following question, which was analyzed among participants who were ≥25 years and reported they were likely to use a future over-the-counter pill: “If you were to use an over-the-counter pill, would you get preventive health screenings, like a Pap smear or testing for sexually transmitted infections, on a regular basis?” We limited these preventive screening questions to participants ≥25 years of age to reflect cervical cancer screening guidelines.^15^

Past difficulties getting birth control were assessed with two questions. First, participants were asked, “Before joining the [ACCESS] study, did you ever try to get a prescription for birth control (like the pill, patch, or ring)?” If they answered “yes” they were then asked, “Before joining the [ACCESS] study, was it ever difficult for you to get a prescription for birth control (like the pill, patch, or ring)?” If they answered “yes” they were asked to specify what challenges they experienced from a list of response options derived from prior research^16^ and an “other—specify” option; participants could select more than one response.

Participants were asked about their birth control use in the month before joining the ACCESS study, and we categorized method use by the most effective method used, excluding emergency contraception.^17^ Race and ethnicity were self-classified by participants using the following questions: “Are you of Spanish, Hispanic, or Latina descent?” and “What is your race? Please mark all that apply.” For the latter question, respondents could mark all that applied from the following options: Asian/Pacific Islander, Black/African American, Native American/Alaska Native, and White/Caucasian. Participants were also asked to report their age, highest level of education and current relationship status (adults only), prior pregnancies, region, whether they had enough money to meet their basic needs in the prior month, current health insurance status, current employment and student status, and ever use of a progestin-only pill before the ACCESS study.

Data analyses were conducted using Stata Statistical Software version 15.1 (StataCorp LLC, College Station, TX) and R: A Language and Environment for Statistical Computing statistical software (R Core Team, Vienna, Austria). We computed descriptive statistics and 2-sided Exact (Clopper-Pearson) 95% confidence intervals (CIs) around proportions for our primary variable of interest on likely over-the-counter pill use. We conducted Pearson's chi-square and Fisher's exact tests to estimate likelihood of over-the-counter progestin-only pill use by background characteristics and prior prescription contraception access barriers, and to compare adult/teen reasons for interest/noninterest and situations for use.

We constructed separate multivariable logistic models assessing whether age, education, race/ethnicity, marital status, prior pregnancies, and insurance status were related to likely over-the-counter progestin-only pill use. We selected these variables because we hypothesized they might have an impact on over-the-counter pill use with potential policy, advocacy, and practice implications. We hypothesized that interest in future use might vary by race/ethnicity due to experiences with racism when accessing health care, which might make Black, Indigenous, and People of Color participants more interested in continuing over-the-counter use. Using web-based software, DAGitty version 3.0 (DAGitty, Nijmegen, The Netherlands), we constructed Directed Acyclic Graphs to identify potential confounding factors for each predictor of interest (Supplementary Appendix Table SA1).

Directed Acyclic Graphs are graphical representations of causal effects between variables, and are used to help choose which covariates should be included in statistical analyses to minimize bias in the estimate produced.^18^ All background characteristics in Table 1 were candidates for model inclusion; unobserved variables were included in the Directed Acyclic Graphs to represent hypothesized pathways. For each model, we included the minimal sufficient adjustment set of variables for estimating the total effect of our predictors of interest on likely over-the-counter progestin-only pill use. There were no potential confounders in the relationships between age or race/ethnicity and likely over-the-counter progestin-only pill use, so we ran unadjusted models for these predictors. Education, marital status, prior pregnancies, and insurance status were assessed among adults only because some variables in these models were asked only among adults.

**Table 1.** Sample Characteristics and Likelihood of Future Use of an Over-the-Counter Progestin-Only Pill, Among People Who Participated in the ACCESS Study, *N* = 665

	Total					Adults					Teens				
	Sample		% Likely to use an over-the-counter progestin-only pill^a^		***p*** ^b^	Sample		% Likely to use an over-the-counter progestin-only pill^a^		***p*** ^b^	Sample		% Likely to use an over-the-counter progestin-only pill^a^		***p*** ^b^
	***n***	%	***n***	%		***n***	%	***n***	%		***n***	%	***n***	%	
All	665	100	553	83		550		458	83		115		95	83	
Age (in years)					0.39					0.16					1.00
11–14	26	4	22	85		—	—	—	—		26	23	22	85	
15–17	89	13	73	82		—	—	—	—		89	77	73	82	
18–24	208	31	169	81		208	38	169	81		—	—	—	—	
25–34	211	32	173	82		211	38	173	82		—	—	—	—	
35–44	116	17	101	87		116	21	101	87		—	—	—	—	
45–60	15	2	15	100		15	3	15	100		—	—	—	—	
Missing	0	0	0	0		0	0	0	0		—	—	—	—	
Highest level of education completed (adults only)										**0.03**					
<High school	—	—	—	—		26	5	22	85		—	—	—	—	
High school graduate	—	—	—	—		107	20	91	85		—	—	—	—	
Some college	—	—	—	—		193	35	171	89		—	—	—	—	
College graduate	—	—	—	—		206	38	160	78		—	—	—	—	
Prefer not to answer	—	—	—	—		2	0.4	2	100		—	—	—	—	
Missing	—	—	—	—		16	3	12	75		—	—	—	—	
Race/ethnicity					**0.01**					**0.01**					0.51
Asian-Pacific Islander, non-Hispanic/Latinx	29	4	24	83		24	4	21	88		5	4	3	60	
Black, non-Hispanic/Latinx	148	22	128	86		125	23	108	86		23	20	20	87	
Hispanic/Latinx	120	18	108	90		101	18	91	90		19	17	17	89	
Native American/Alaska Native, non-Hispanic/Latinx	4	1	2	50		4	1	2	50		0	0	0	0	
White, non-Hispanic/Latinx	289	43	226	78		236	43	184	78		53	46	42	79	
Two or more races, non-Hispanic/Latinx	38	6	34	89		29	5	27	93		9	8	7	78	
Missing	37	6	31	84		31	6	25	81		6	5	6	100	
Current relationship status (adults only)										0.63					
Married	—	—	—	—		112	20	92	82		—	—	—	—	
Divorced/widowed/separated	—	—	—	—		28	5	26	93		—	—	—	—	
Never married, living alone	—	—	—	—		268	49	223	83		—	—	—	—	
Never married, living with partner	—	—	—	—		121	22	101	83		—	—	—	—	
Prefer not to answer	—	—	—	—		5	1	4	80		—	—	—	—	
Missing	—	—	—	—		16	3	12	75		—	—	—	—	
Prior pregnancies					**0.003**					**0.001**					0.32
Yes	290	44	255	88		284	52	251	88		6	5	4	67	
No	352	53	279	79		247	45	192	78		105	91	87	83	
Prefer not to answer	3	0.5	3	100		3	1	3	100		0	0	0	0	
Missing	20	3	16	80		16	3	12	75		4	3	4	100	
Region					0.30					0.35					0.87
Northeast	94	14	76	81		69	13	55	80		25	22	21	84	
Midwest	65	10	50	77		48	9	37	77		17	15	13	76	
South	314	47	269	86		275	50	236	86		39	34	33	85	
West	169	25	139	82		139	25	115	83		30	26	24	80	
Missing	23	3	19	83		19	4	15	79		4	3	4	100	
During the past month, would you say you had enough money to meet your basic living needs, such as food, housing, and transportation?					0.94					0.63					0.22
All or most of the time	436	66	363	83		356	65	300	84		80	70	63	79	
Sometimes, rarely, or never	176	26	147	84		155	28	128	83		21	18	19	90	
Do not know	7	1	5	71		5	1	3	60		2	2	2	100	
Prefer not to answer	22	3	18	82		15	3	12	80		7	6	6	86	
Missing	24	4	20	83		19	4	15	79		5	4	5	100	
Current health insurance					0.09					**0.04**					0.59
Public	215	32	189	88		184	34	163	89		31	27	26	84	
Private	232	35	184	79		195	36	152	78		37	32	32	86	
Other	3	1	3	100		3	1	3	100		0	0	0	0	
None	109	16	92	84		102	19	87	85		7	6	5	71	
Do not know	55	8	46	84		23	4	21	91		32	28	25	78	
Prefer not to answer	29	4	21	72		25	5	18	72		4	4	3	75	
Missing	22	3	18	82		18	3	14	78		4	4	4	100	
Current employment					0.15					0.19					0.25
Yes	370	56	303	82		338	62	279	83		32	28	24	75	
No	254	38	219	86		177	32	154	87		77	67	65	84	
Prefer not to answer	21	3	15	71		19	4	13	68		2	2	2	100	
Missing	20	3	16	80		16	3	12	75		4	4	4	100	
Current student status					0.45					0.61					1.00
Yes	278	42	228	82		171	31	141	82		107	93	87	81	
No	356	54	300	84		355	65	299	84		1	1	1	100	
Prefer not to answer	11	2	9	82		8	2	6	75		3	3	3	100	
Missing	20	3	16	80		16	3	12	75		4	4	4	100	
Most effective birth control method used in the month before joining the ACCESS study					0.26					0.13					0.84
Ring, patch, injectable, implant, intrauterine device, vasectomy	43	6	37	86		38	7	33	87		5	4	4	80	
Oral contraceptive	104	16	80	77		94	17	71	76		10	9	9	90	
Less effective method	115	17	95	83		103	19	86	84		12	10	9	75	
No method	366	55	311	85		287	52	246	86		79	69	65	82	
Missing	37	6	30	81		28	5	22	79		9	8	8	89	
Ever used any progestin-only pill before the ACCESS study					0.31					0.26					0.61
Yes	78	12	69	89		75	14	67	89		3	3	2	67	
No	465	70	386	83		373	68	310	83		92	80	76	83	
Not sure	105	16	84	80		90	16	72	80		15	13	12	80	
Prefer not to answer	7	1	7	100		4	1	4	100		3	3	3	100	
Missing	10	2	7	70		8	2	5	63		2	2	2	100	

^a^ Participants were considered likely to use an over-the-counter progestin-only pill if they reported being very likely or somewhat likely (vs. somewhat unlikely, very unlikely, not sure, or did not answer).

^b^ Assessed via chi-square and Fisher's exact tests, which excluded “Missing” and “Prefer not to answer” responses. Bolded *p*-values indicate significance of *p* < 0.05.

“—”: Data not analyzed in this population.

ACCESS, Adherence with Continuous Dose Oral Contraceptive: Evaluation of Self-Selection and Use.

For education, we controlled for age and race/ethnicity; to assess for possible bias on education by age, we also ran a model for education that was restricted to participants 25 years of age or older and who were therefore more likely to have had a chance to complete college, as well as a model with an interaction term testing whether the effect of education differed by age. For marital status, we controlled for age and education; for prior pregnancies we controlled for age, education, and marital status; and for insurance status, we controlled for marital status, income (*i.e*., whether they had enough money to meet basic needs in the prior month), employment status, and student status.

We included missing data as a covariate in tables; we excluded “Missing” and “Prefer not to answer” responses in Pearson's chi-square and Fisher's exact tests and regression analyses. Open-ended text was coded in Excel using an inductive process. Individual responses were reviewed to discover underlying themes in the data. For categorical questions with an open response option, we recoded open response text to the appropriate category if it reflected an existing response option and created new response categories for emerging themes. Illustrative quotes presented in this article are identified using participants' age and region. The STROBE checklist for cross-sectional studies^19^ was used in reporting our findings.

## Results

Among the 883 individuals invited from the ACCESS study, 665 took part in our survey (75% response rate), including 550 adults (81% of ACCESS study adults) and 115 teens (58% of ACCESS study teens). Participants completed the survey in a median time of 10 minutes. See Table 1 for participant characteristics.

Overall, 83% of both adults (95% CI: 79.9–86.3, including 59.0% very likely and 24.4% somewhat likely) and teens (95% CI: 74.4–89.0, including 45.2% very likely and 37.4% somewhat likely) reported likely future over-the-counter progestin-only pill use if available. The proportions of participants who were likely to use an over-the-counter progestin-only pill are presented by background characteristic in Table 1.

In the logistic regression models, neither age nor marital status were associated with likelihood of future over-the-counter progestin-only pill use. In the models for race/ethnicity, education, prior pregnancies, and insurance status, Hispanic/Latinx (odds ratio [OR] = 2.51, 95% CI: 1.30–4.85, *p* = 0.006) and Black participants (OR = 1.78, 95% CI: 1.03–3.09, *p* = 0.04) (vs. White), and adults with some college (adjusted odds ratio [AOR] = 2.34, 95% CI: 1.30–4.18, *p* = 0.004) (vs. college degree), prior pregnancies (AOR = 1.90, 95% CI: 1.08–3.34, *p* = 0.03) (vs. none), and public insurance (AOR = 2.67, 95% CI: 1.42–5.03, *p* = 0.002) (vs. private) were significantly more likely to report likely future over-the-counter progestin-only pill use (Supplementary Appendix Table SA1).

In the model we ran for education that was restricted to participants 25 years of age and older, the results were consistent, with those with some college (vs. college degree) significantly more likely to report likely future over-the-counter progestin-only pill use. However, when examining the effects of education by age group, we found that the education effect whereby participants with some college were significantly more likely to report likely future over-the-counter progestin-only pill use than those with a college degree was concentrated solely among 25–34 year olds.

Among likely users of an over-the-counter progestin-only pill, most adults and teens (90% each) reported interest in using the pill for as long as birth control was needed, as opposed to as a short-term bridge to another method. There were no statistical differences between adults and teens in the situations in which participants would use an over-the-counter progestin-only pill (Supplementary Appendix Table SA2). Both interested adults and teens reported that they would pay up to a median price of $20 per month (Supplementary Appendix Table SA3).

Primary reasons for interest among adults and teens (*n* = 553) included convenience (81%), ease of access (80%), saving time (77%) and money (64%) not to have to visit a clinic, the ability to get it when traveling (59%), someone else could get it (49%), and greater privacy (42%). Trends were largely similar for adults and teens, although a significantly larger proportion of teens reported privacy as a reason for interest (*p* = 0.02), and a smaller proportion of teens reported they could send someone else to get their birth control (*p* = 0.002). Additionally, a smaller proportion of teens reported not having insurance as a reason for interest, compared with adults (*p* < 0.001) (Table 2).

**Table 2.** Reasons Participants Were Interested in Using an Over-the-Counter Progestin-Only Pill, Among Those Likely to Use an Over-the-Counter Pill

Reasons for interest	Total (***n*** = 553)	Adult (***n*** = 458)	Teen (***n*** = 95)	***p*** ^a^
	***n* (%)**	***n* (%)**	***n* (%)**	
It would be more convenient	447 (80.8)	373 (81.4)	74 (77.9)	0.42
It would be easier to get birth control	444 (80.3)	373 (81.4)	71 (74.7)	0.14
It would save time to not have to visit a doctor or nurse	427 (77.2)	361 (78.8)	66 (69.5)	0.05
It would be easier to get a pack of pills whenever I run out	402 (72.7)	339 (74.0)	63 (66.3)	0.13
It would save money to not have to pay for a visit to the doctor or nurse	356 (64.4)	301 (65.7)	55 (57.9)	0.15
I can get it when I am traveling or away from home	326 (59.0)	277 (60.5)	49 (51.6)	0.11
I could send someone else to get my birth control when I needed it	271 (49.0)	238 (52.0)	33 (34.7)	**0.002**
It would feel more private or I could get it without others knowing	233 (42.1)	183 (40.0)	50 (52.6)	**0.02**
I don't want to get a physical or pelvic exam to get birth control pills	219 (39.6)	182 (39.7)	37 (38.9)	0.89
I don't have insurance or my insurance does not cover birth control	115 (20.8)	108 (23.6)	7 (7.4)	**<0.001**
I don't want to use insurance	57 (10.3)	43 (9.4)	14 (14.7)	0.12
Some other reason	7 (1.3)	6 (1.3)	1 (1.1)	1.00
Prefer not to answer	3 (0.5)	2 (0.4)	1 (1.1)	**-**

More than one response possible.

Participants were considered likely to use an over-the-counter progestin-only pill if they reported being very likely or somewhat likely (vs. somewhat unlikely, very unlikely, not sure, or did not answer).

^a^ Assessed via chi-square and Fisher's exact tests. Bolded *p*-values indicate significance of *p* < 0.05.

“—”: Data not analyzed in this population.

In an open response comment box asking participants to tell us more about why they were likely to use an over-the-counter progestin-only pill, respondents expounded on how it would afford greater convenience and access. As one participant described, “It's very inconvenient to have to get birth control from my physician. First, I have to make an appointment, which can be months away…. Then I have to get an annual pap before she will renew my existing prescription. It's such a hassle. Much easier to get the pill over the counter” (age 42, South). One participant explained how being able to pick the pill up at the store would give her greater control in preventing pregnancy: “Because having to go to a Dr to protect myself from not having another baby when I'm not ready is way harder then *[sic]* just grabbing it at the store when I'm checking out” (age 29, West). Other respondents described the convenience of being able to get the pill on short notice to avoid running out of pills (age 35, West).

Respondents also discussed positive side effects (such as reduced periods) or a lack of side effects while using the progestin-only pill as contributors to their interest, as well as feeling the product was effective at preventing pregnancy and easy to use. As one participant stated, “It is easy to use. Simply take daily. There were no noticeable changes or side effects” (age 23, Midwest). Another participant liked that she did not have a period at all while taking the pill: “Easy to follow. No major side effects beside not having a period. Think it would be a good benefit to have on top of other protection” (age 35, Midwest). For others, not having health insurance was the primary driver, such as one participant who said, “I'm a poor, uninsured millennial” (age 33, South) and another who shared, “Lack of insurance due to job loss from covid” (age 37, West).

Young people who reported privacy as a motivator cited the ability to make their own birth control decisions without asking or telling their parents. As one teen reported, “Because [birth control is] important to have but I don't want to have to ask or tell my parents, it should be my choice” (age 14, West). Another teen stated, “I am uncomfortable talking to my parents about topics like sex, and although I know it would be kept confidential with my doctor, I am afraid my parents would find out by pressuring me to tell them if they ever found out about any unknown doctor visits. I am safe, knowledgeable about the risk of sexually transmitted diseases, I would just use [birth control] to live my life and be safe, preventing any pregnancy or abortion in my life” (age 16, West).

Primary reasons for noninterest in using an over-the-counter progestin-only pill among adults and teens (*n* = 86) included the bleeding associated with progestin-only pill use (52%), not being interested in a progestin-only pill (36%), wanting a health care provider to make sure the pill is right for them (33%), and concern about over-the-counter pills being more expensive than prescription pills (26%). Only concerns about bleeding was statistically different (*i.e*., higher) among teens compared with adults (*p* = 0.03) (Table 3).

**Table 3.** Reasons Participants Were Not Interested in Using an Over-the-Counter Progestin-Only Pill, Among Those Not Likely to Use an Over-the-Counter Pill

Reasons for noninterest	Total (***n*** = 86)	Adult (***n*** = 72)	Teen (***n*** = 14)	***p*** ^a^
	***n* (%)**	***n* (%)**	***n* (%)**	
I did not like the bleeding that I experienced with this progestin-only birth control pill	45 (52.3)	34 (47.2)	11 (78.6)	**0.03**
I am not interested in a progestin-only birth control pill	31 (36.1)	24 (33.3)	7 (50.0)	0.24
I want a doctor or nurse to make sure the pill is right for me	28 (32.6)	21 (29.2)	7 (50.0)	0.21
The cost of this over-the-counter pill may be higher than the prescription-only pill	22 (25.6)	18 (25.0)	4 (28.6)	0.75
I am concerned about how effective this pill is	14 (16.3)	11 (15.3)	3 (21.4)	0.69
I am concerned I won't be able to take the pill at the right time each day	13 (15.1)	11 (15.3)	2 (14.3)	1.00
Other side effects	8 (9.3)	8 (11.1)	0 (0.0)	0.34
I am not interested in any kind of birth control pill	7 (8.1)	7 (9.7)	0 (0.0)	0.59
I might not use the pill correctly if I don't talk to a doctor or nurse	1 (1.2)	1 (1.4)	0 (0.0)	1.00
Prefer not to answer	0 (0.0)	0 (0.0)	0 (0.0)	**-**

More than one response possible.

Participants were considered not likely to use an over-the-counter progestin-only pill if they reported being very unlikely or somewhat unlikely (vs. somewhat likely, very likely, not sure, or did not answer).

^a^ Assessed via chi-square and Fisher's exact tests. Bolded *p*-values indicate significance of *p* < 0.05.

“—”: Data not analyzed in this population.

In an open response comment box asking participants to tell us more about why they were not likely to use an over-the-counter progestin-only pill, participants elaborated on the negative bleeding they experienced, citing irregular periods, heavier bleeding cycles, and/or cramping; a few respondents reported undesired amenorrhea or conversely wished that the pill had caused amenorrhea. As one respondent stated, “It wasn't the right birth control for me, but it didn't cause any major problems. Just made my periods irregular, which is the most common side effect for progesterone-only birth control” (age 33, Northeast). Another reported, “I only take birth control to not have a period and it didn't prevent a period” (age 28, West). A few also experienced other side effects such as weight gain or headaches that contributed to their noninterest. Regarding concerns about cost, most cited that they could get birth control for free through their health insurance, and thought they would have to pay out of pocket for an over-the-counter pill.

As one respondent stated, “Birth control is covered by my insurance, so I do not have to pay…. Why spend money when I do not have to?” (age 23, South). Preference for another contraceptive method was also cited as a reason for noninterest for some, including wanting a long-acting method or a method that they did not need to take daily.

Overall, 75% of participants ≥25 years of age reported they had had a cervical cancer screening in the prior 3 years. Among those ≥25 years of age and who reported likely over-the-counter progestin-only pill use, 79% reported they were likely to get future preventive health screenings on a regular basis.

Among those who had ever tried to access prescription birth control (pill, patch, or ring) before the ACCESS study (*n* = 355), 43% reported having difficulties getting it, including 44% of adults and 29% of teens. Participants who experienced challenges were more likely to report interest in using an over-the-counter progestin-only pill (89%) compared with those who did not face challenges (79%) (*p* = 0.01).

Top challenges getting prescription birth control among those who had ever tried included difficulty getting an appointment (22%), difficulty paying for the method (21%), not having a regular doctor or clinic (21%), clinic hours being inconvenient (19%), difficulty paying for an appointment (19%), being uninsured (18%), difficulty getting childcare or time off from work or school (18%), not wanting a physical or pelvic exam (14%), and difficulty getting to a clinic (13%). A significantly larger proportion of adults compared with teens reported not having a regular doctor or clinic (*p* = 0.04), difficulty paying for an appointment (*p* = 0.02), and being uninsured (*p* = 0.03) as challenges (Table 4).

**Table 4.** Challenges Accessing Prescription Birth Control (Pill, Patch, or Ring), Among Those Who Had Ever Tried

Challenges	Total (***n*** = 355)	Adult (***n*** = 324)	Teen (***n*** = 31)	***p*** ^a^
	***n* (%)**	***n* (%)**	***n* (%)**	
It was hard to get an appointment	77 (21.7)	73 (22.5)	4 (12.9)	0.21
It was difficult to pay for birth control or my insurance wouldn't cover it	76 (21.4)	73 (22.5)	3 (9.7)	0.10
I did not have a regular doctor or clinic	74 (20.8)	72 (22.2)	2 (6.5)	**0.04**
Doctor or clinic office hours are not convenient	68 (19.2)	64 (19.8)	4 (12.9)	0.36
It was difficult to pay for an appointment at a clinic	66 (18.6)	65 (20.1)	1 (3.2)	**0.02**
I did not have insurance	64 (18.0)	63 (19.4)	1 (3.2)	**0.03**
It was hard to get time off from work, school, or childcare	64 (18.0)	59 (18.2)	5 (16.1)	0.77
I did not want to have a physical exam or pelvic exam	49 (13.8)	47 (14.5)	2 (6.5)	0.28
It was hard to get to a clinic	45 (12.7)	43 (13.3)	2 (6.5)	0.40
It was hard to get to a pharmacy	23 (6.5)	22 (6.8)	1 (3.2)	0.71
Hard to get preferred method from doctor	5 (1.4)	5 (1.5)	0 (0)	1.00
Didn't want parents to know	4 (1.1)	3 (0.9)	1 (3.2)	0.31
Other	6 (1.7)	5 (1.5)	1 (3.2)	0.42
Missing	1 (0.3)	1 (0.3)	0 (0)	—

More than one response possible.

^a^ Assessed via chi-square and Fisher's exact tests. Bolded *p*-values indicate significance of *p* < 0.05.

“—”: Data not analyzed in this population.

## Discussion

Most adults and teens (83%) in the ACCESS study reported they would likely continue to use an over-the-counter progestin-only pill if it were available, and Hispanic/Latinx and Black participants as well as adults with public insurance, prior pregnancies, and some college had higher likelihood of interest compared with their counterparts. Prior experience of barriers to birth control access also contributed to participants' interest in over-the-counter pill use. These findings, among people who used a progestin-only pill in an over-the-counter environment during the ACCESS study, suggest higher interest than what has been found in prior research, where 39% of adults at risk of unintended pregnancy and 29% of teens reported likely use of an over-the-counter progestin-only pill if available in one study,^13^ and 37% of adults at risk of unintended pregnancy reported the likely use of an over-the-counter oral contraceptive in another earlier study.^14^

The prior research on over-the-counter progestin-only pill interest^13^ was limited in that few oral contraceptive users in the United States use progestin-only pills,^11^ so responses were largely hypothetical. Our study included people who had actually used a progestin-only pill, and demonstrated that interest in continued over-the-counter use was high. The higher interest in our study likely reflects the motivation inherent in our study population who had already elected to use an over-the-counter pill during the ACCESS study. It also highlights the real-world acceptability of taking a progestin-only pill without a prescription among likely users.

Among those in our study who were not interested in using an over-the-counter progestin-only pill in the future, the most common reason was not liking the bleeding they had experienced. Like all progestin-only methods, progestin-only pills cause bleeding changes in a significant proportion of users, and the most common complaint among progestin-only pill users in general is irregular bleeding.^20,21^ At the same time, some participants in our study considered the bleeding changes they experienced a benefit of the method, which is also reflected among progestin-only pill users more broadly,^20^ highlighting people's differing contraceptive preferences and experiences. These data indicate the need for an education campaign to inform potential users about possible bleeding changes if progestin-only pills become available over the counter.

Our findings on the high likelihood of future preventive health screenings among individuals likely to use an over-the-counter progestin-only pill echo prior research on the United States/Mexico border, which found a high proportion of individuals who got oral contraceptives over the counter in Mexico obtained recent preventive health screenings.^8^

### Limitations

Our study has several limitations. Our sample included people who had participated in the ACCESS study, and this population may not reflect the general population of over-the-counter pill users. Their background characteristics are not necessarily reflective of all potential users, and trial participants may be more highly motivated to use an over-the-counter pill compared with individuals who do not participate in clinical trial research; it is also possible that our study may underestimate interest because people most likely not to have health care and/or who may need easier access might not know about or join clinical trials. Finally, we were not able to compare the background characteristics of those who participated in our survey with the broader ACCESS study participants, and while we had a high response rate, there may be additional biases on experiences with the pill that cause selection bias for our survey. Despite these limitations, these data capture user experiences of an over-the-counter progestin-only pill for the first time.

## Conclusions

These findings document high interest in over-the-counter progestin-only pill use among individuals who had used an over-the-counter pill in a study environment. These data highlight the real-world acceptability of taking a progestin-only pill without a prescription among likely users in the United States, and provide rich insights into user experiences that can inform efforts for over-the-counter access. This study shows that coupled with high acceptability, over-the-counter access could increase access to birth control for a large group of people in the United States.

## Supplementary Material

Supplemental data

Supplemental data

Supplemental data

## Supplementary Material

Supplementary Appendix Table SA1

Supplementary Appendix Table SA2

Supplementary Appendix Table SA3

## Abbreviations Used

ACCESS: Adherence with Continuous Dose Oral Contraceptive: Evaluation of Self-Selection and Use
AOR: adjusted odds ratio
CI: confidence interval
FDA: Food and Drug Administration
OR: odds ratio
