# Supplementary material for: Interest in Continued Use After Participation in a Study of Over-the-Counter Progestin-Only Pills in the United States
Source: Womens Health Rep (New Rochelle). 2022 Nov 9;3(1):904–14. doi: 10.1089/whr.2022.0056 (PMC9712042; doi:10.1089/whr.2022.0056)
Supplement: Supplemental data [file Supp_AppendixTableS1.docx]

**Supplemental Appendix 1: Directed Acyclic Graphs to identify potential confounders related to likely over-the-counter progestin-only pill use in models for age, education, race/ethnicity, marital status, prior pregnancies, and insurance status**

Legend:


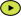
exposure **– predictor of interest**


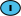
outcome **– outcome of interest**

adjusted variable **– confounder variable included in logistic regression analysis**


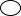


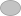
**other variable – variable not a confounder and not included in logistic regression analysis**

unobserved (latent) **– unobserved variable representing hypothesized pathway**


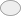


**Model A: Age**

**
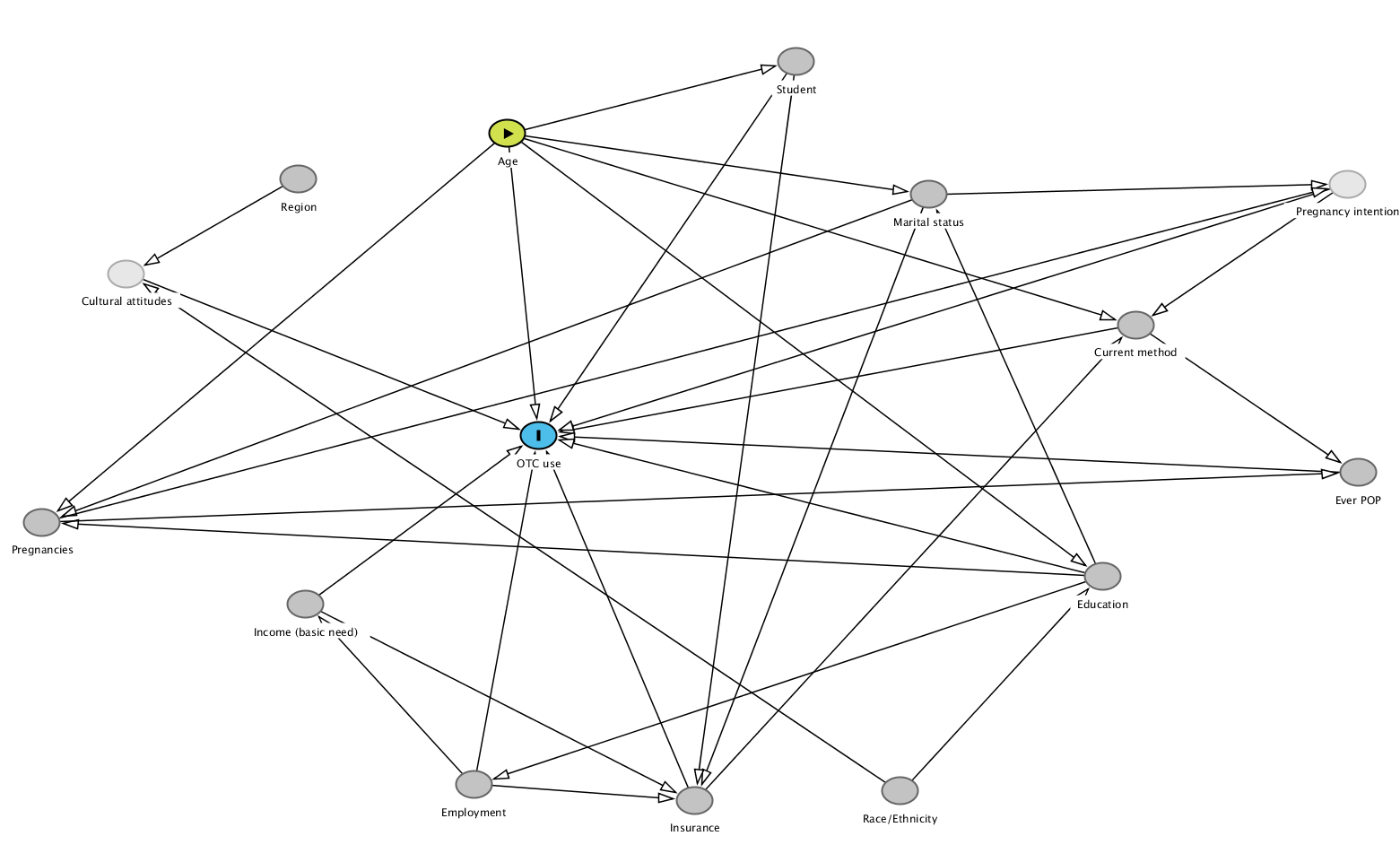
**

|  | **Likely to use an over-the-counter progestin-only pill*** | | |
| --- | --- | --- | --- |
| **Age (in years)** | **Odds ratio** | **95% confidence interval** | |
| **11-14** | **1.21** | **0.39** | **3.71** |
| **15-17** | **1.00** | **0.53** | **1.91** |
| **18-24** | **0.95** | **0.58** | **1.56** |
| **25-34** | ***Ref*** |  |  |
| **35-44** | **1.48** | **0.78** | **2.82** |
| **45-60** | **1.00** |  |  |
| ***No adjustment was necessary to estimate the total effect of age on likely over-the-counter progestin-only pill use.***  ***Participants were considered likely to use an over-the-counter progestin-only pill if they reported being very likely or somewhat likely (vs. somewhat unlikely, very unlikely, not sure, or did not answer).** | | | |

**Model B: Education**

**
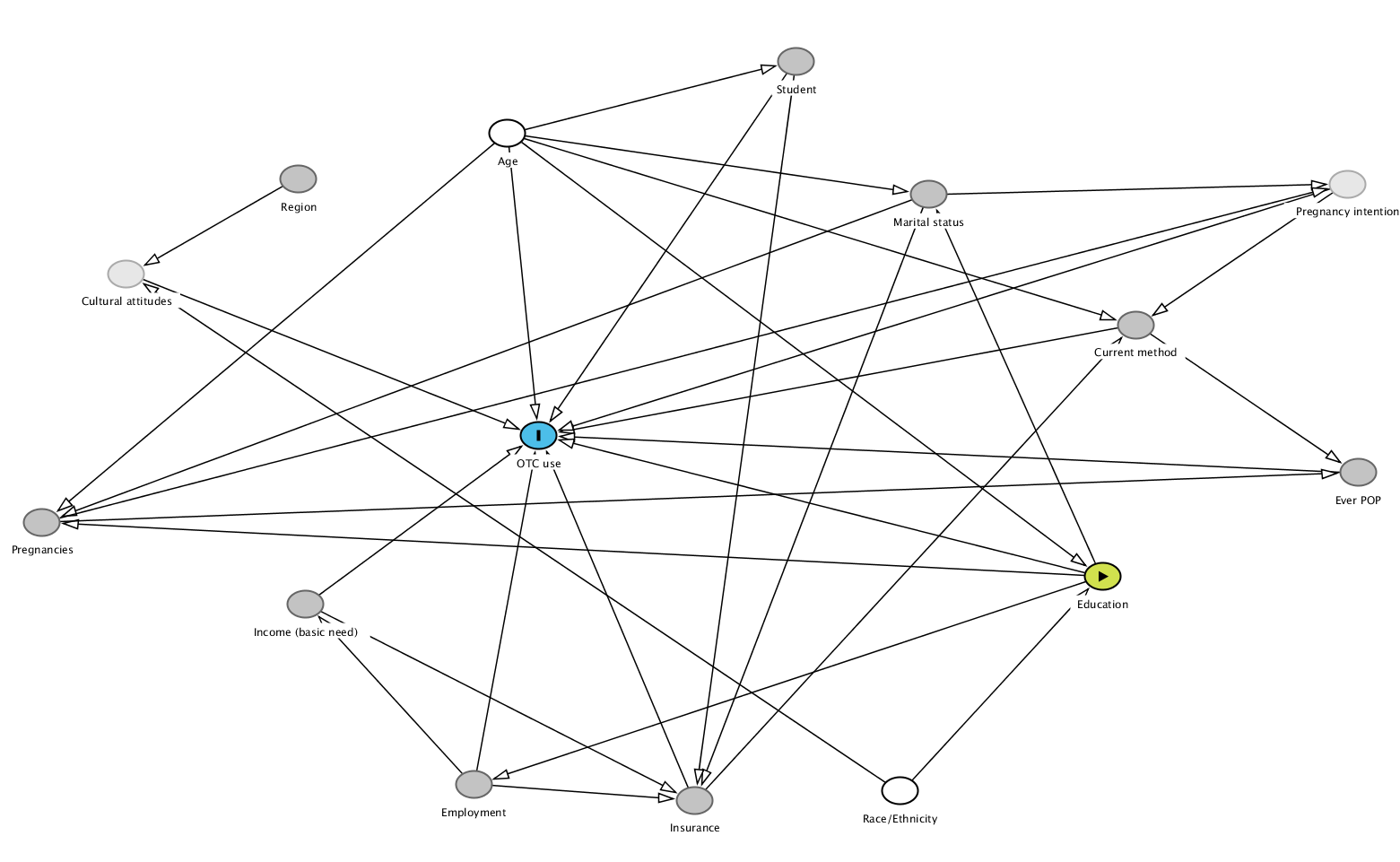
**

|  | **Likely to use an over-the-counter progestin-only pill*** | | |
| --- | --- | --- | --- |
| **Highest level of education completed (adults only)** | **Adjusted odds ratio** | **95% confidence interval** | |
| **<High school** | **1.45** | **0.45** | **4.65** |
| **High school graduate** | **1.61** | **0.83** | **3.13** |
| **Some college** | **2.34** | **1.30** | **4.18** |
| **College graduate** | ***Ref*** |  |  |
| ***Controlling for age and race/ethnicity.***  ***Participants were considered likely to use an over-the-counter progestin-only pill if they reported being very likely or somewhat likely (vs. somewhat unlikely, very unlikely, not sure, or did not answer).** | | | |

**Model C: Race/ethnicity**

**
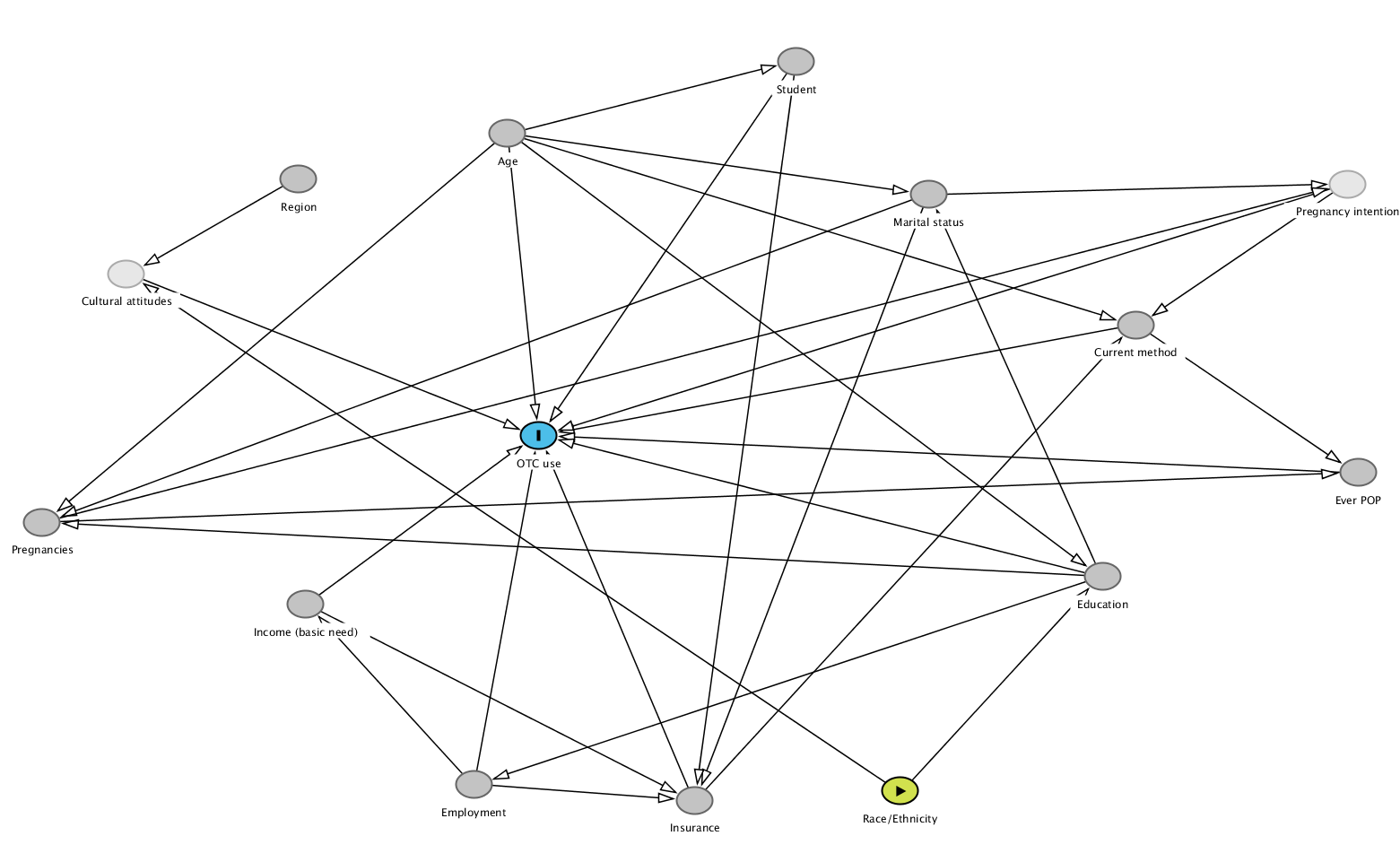
**

|  | **Likely to use an over-the-counter progestin-only pill*** | | |
| --- | --- | --- | --- |
| **Race/ethnicity** | **Odds ratio** | **95% confidence interval** | |
| **Asian-Pacific Islander, non-Hispanic/Latinx** | **1.34** | **0.49** | **3.65** |
| **Black, non-Hispanic/Latinx** | **1.78** | **1.03** | **3.09** |
| **Hispanic/Latinx** | **2.51** | **1.30** | **4.85** |
| **Native American/Alaska Native, non-Hispanic/Latinx** | **0.28** | **0.04** | **2.02** |
| **White, non-Hispanic/Latinx** | ***Ref*** |  |  |
| **Two or more races, non-Hispanic/Latinx** | **2.37** | **0.81** | **6.93** |
| ***No adjustment was necessary to estimate the total effect of race/ethnicity on likely over-the-counter progestin-only pill use.***  ***Participants were considered likely to use an over-the-counter progestin-only pill if they reported being very likely or somewhat likely (vs. somewhat unlikely, very unlikely, not sure, or did not answer).** | | | |

**Model D: Marital status
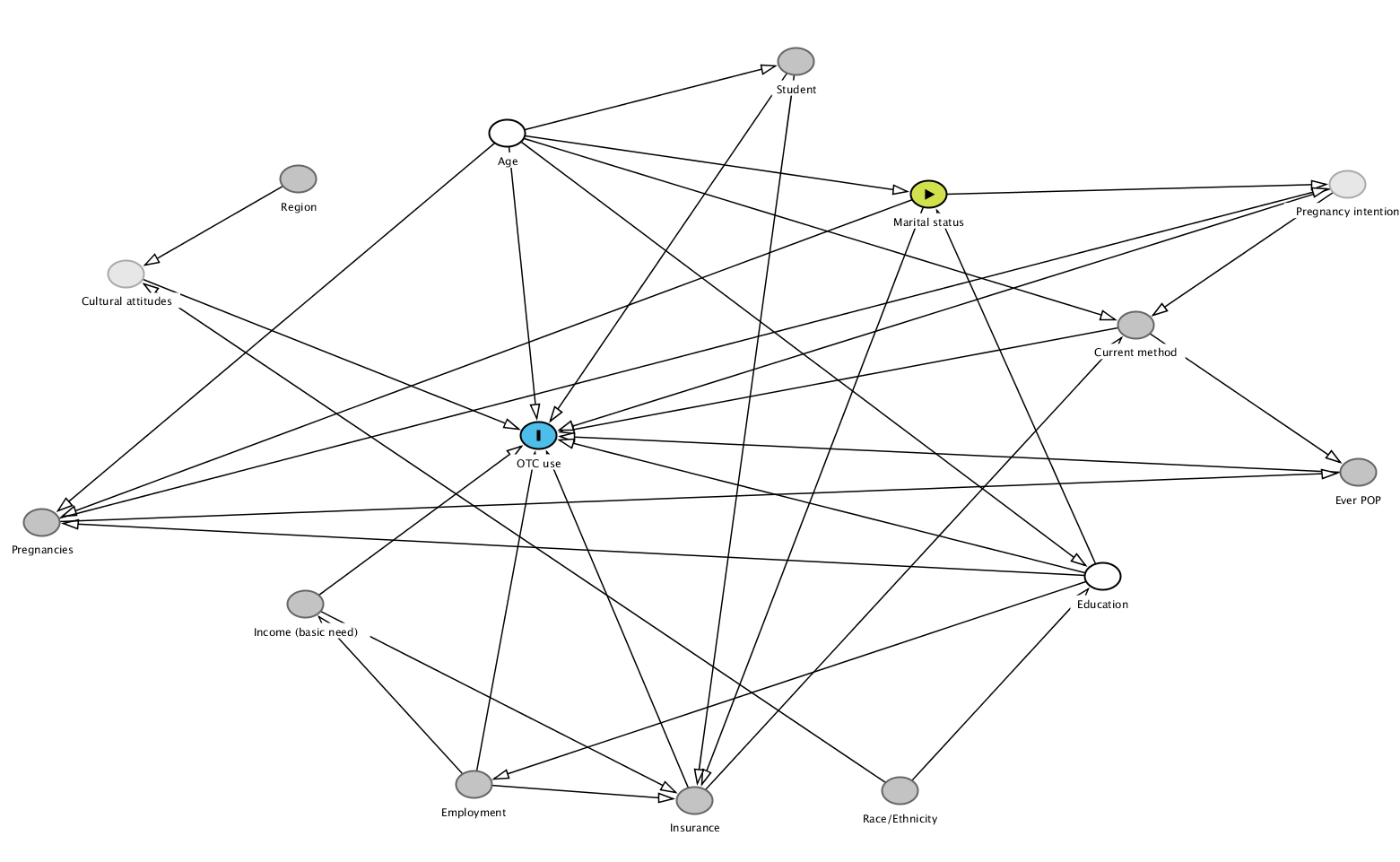
**

|  | **Likely to use an over-the-counter progestin-only pill*** | | |
| --- | --- | --- | --- |
| **Current relationship status (adults only)** | **Adjusted odds ratio** | **95% confidence interval** | |
| **Married** | **0.84** | **0.45** | **1.55** |
| **Divorced/ widowed/ separated** | **1.60** | **0.35** | **7.38** |
| **Never married, living alone** | ***Ref*** |  |  |
| **Never married, living with partner** | **0.96** | **0.53** | **1.75** |
| ***Controlling for age and education.***  ***Participants were considered likely to use an over-the-counter progestin-only pill if they reported being very likely or somewhat likely (vs. somewhat unlikely, very unlikely, not sure, or did not answer).** | | | |

**Model E: Prior pregnancy
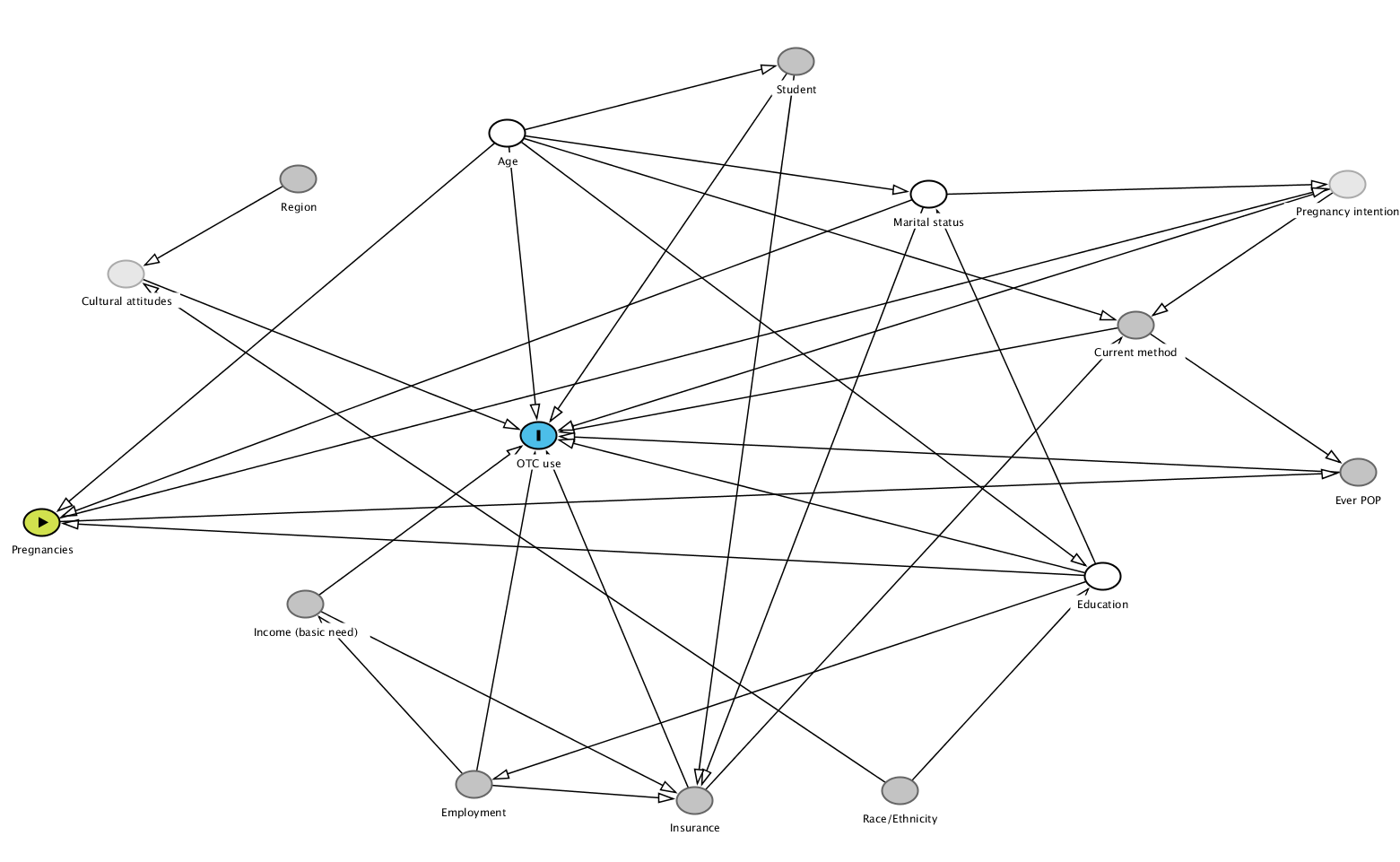
**

|  | **Likely to use an over-the-counter progestin-only pill*** | | |
| --- | --- | --- | --- |
| **Prior pregnancies (adults only)** | **Adjusted odds ratio** | **95% confidence interval** | |
| **No** | ***Ref*** |  |  |
| **Yes** | **1.90** | **1.08** | **3.34** |
| ***Controlling for age, education, and marital status.***  ***Participants were considered likely to use an over-the-counter progestin-only pill if they reported being very likely or somewhat likely (vs. somewhat unlikely, very unlikely, not sure, or did not answer).** | | | |

**Model F: Insurance status
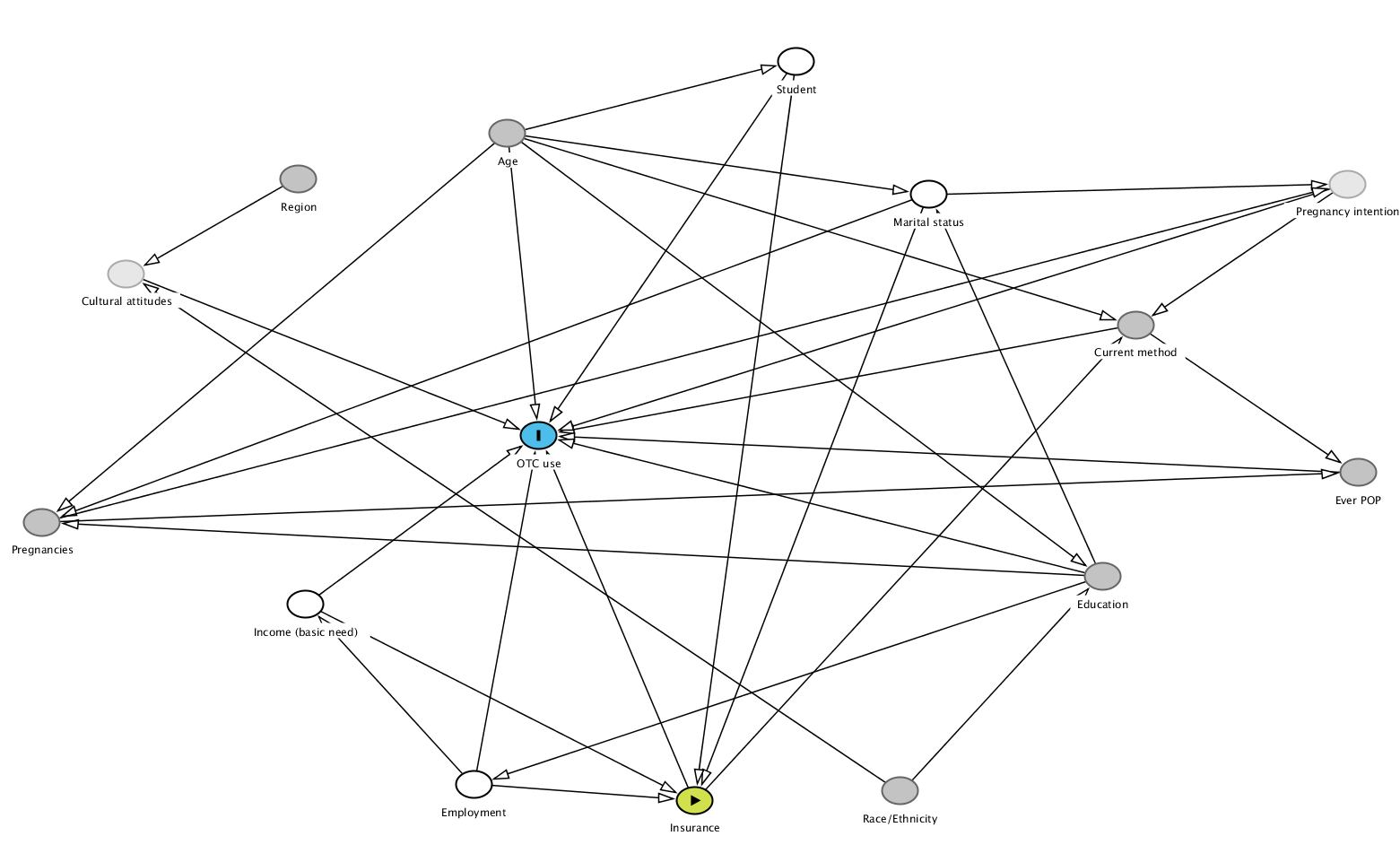
**

|  | **Likely to use an over-the-counter progestin-only pill*** | | |
| --- | --- | --- | --- |
| **Current health insurance (adults only)** | **Adjusted odds ratio** | **95% confidence interval** | |
| **Public** | **2.67** | **1.42** | **5.03** |
| **Private** | ***Ref*** |  |  |
| **Other** | **1.00** |  |  |
| **None** | **2.04** | **0.98** | **4.26** |
| ***Controlling for marital status, income (i.e., whether they had enough money to meet basic needs in the prior month), employment status, and student status.***  ***Participants were considered likely to use an over-the-counter progestin-only pill if they reported being very likely or somewhat likely (vs. somewhat unlikely, very unlikely, not sure, or did not answer).** | | | |
