# Supplementary material for: Interest in Continued Use After Participation in a Study of Over-the-Counter Progestin-Only Pills in the United States
Source: Womens Health Rep (New Rochelle). 2022 Nov 9;3(1):904–14. doi: 10.1089/whr.2022.0056 (PMC9712042; doi:10.1089/whr.2022.0056)
Supplement: Supplemental data [file Supp_AppendixTableS2.docx]

**Supplemental Appendix 2. Situations participants would consider using an over-the-counter progestin-only pill, among those likely to use an over-the-counter pill^*†^**

| **Situations for use** | **Total  (n=553)** | **Adult (n=458)** | **Teen (n=95)** | **p-value**^‡^ | |  |
| --- | --- | --- | --- | --- | --- | --- |
|  | **n (%)** | **n (%)** | **n (%)** |  |  |  |
| I would be interested in staying on it for as long as I need birth control, if I don’t have any problems | 498 (90.1) | 412 (90.0) | 86 (90.5) | **0.87** | |  |
| For short-term use when I run out of prescription birth control pills and can’t get a refill quickly | 127 (23.0) | 104 (22.7) | 23 (24.2) | **0.75** | |  |
| For short-term use before I get onto different birth control method | 96 (17.4) | 84 (18.3) | 12 (12.6) | **0.18** | |  |
| Some other situation | 13 (2.4) | 11 (2.4) | 2 (2.1) | **1.00** | |  |
| If didn't have insurance^∆^ | 4 (0.7) | 4 (0.9) | 0 (0.0) | **1.00** | |  |
| Prefer not to answer | 3 (0.5) | 2 (0.4) | 1 (2.0) | **-** | |  |
| ^*^Participants were considered likely to use an over-the-counter progestin-only pill if they reported being very likely or somewhat likely (vs. somewhat unlikely, very unlikely, not sure, or did not answer). ^†^More than one response possible.  **^‡^Assessed via Chi-square and Fisher's Exact tests.**  ^∆^Recoded from the “Other-Specify” response option.  **- : Data not analyzed in this population.** | | | | |  | |
