# Supplementary material for: Interest in Continued Use After Participation in a Study of Over-the-Counter Progestin-Only Pills in the United States
Source: Womens Health Rep (New Rochelle). 2022 Nov 9;3(1):904–14. doi: 10.1089/whr.2022.0056 (PMC9712042; doi:10.1089/whr.2022.0056)
Supplement: Supplemental data [file Supp_AppendixTableS3.docx]

**Supplemental Appendix 3. Willingness to pay for an over-the-counter progestin-only pill, among those likely to use an over-the-counter pill^*^**

| **Highest price ($) would pay for each month's supply** | **Total  (n=553)** | **Adult (n=458)** | **Teen (n=95)** |
| --- | --- | --- | --- |
|  | **n (%)** | **n (%)** | **n (%)** |
| $0 | 3 (0.5) | 2 (0.4) | 1 (1.1) |
| $1-10 | 143 (25.9) | 126 (27.5) | 17 (17.9) |
| $11-20 | 242 (43.8) | 203 (44.3) | 39 (41.1) |
| $21-30 | 108 (19.5) | 83 (18.1) | 25 (26.3) |
| >$30 | 54 (9.8) | 41 (9.0) | 13 (13.7) |
| Missing | 3 (0.5) | 3 (0.7) | 0 (0.0) |
| Mean price (among participants willing to pay >$0 only) | $20.54 | $20.02 | $23.08 |
| Median price (among participants willing to pay >$0 only) | $20.00 | $20.00 | $20.00 |
| ^*^Participants were considered likely to use an over-the-counter progestin-only pill if they reported being very likely or somewhat likely (vs. somewhat unlikely, very unlikely, not sure, or did not answer). | | | |
